# Comparative Analysis of the Therapeutic Potential of Extracellular Vesicles Secreted by Aged and Young Bone Marrow‐Derived Mesenchymal Stem Cells in Osteoarthritis Pathogenesis

**DOI:** 10.1111/cpr.13776

**Published:** 2024-12-20

**Authors:** Shital Wakale, Yang Chen, Antonia Rujia Sun, Chamikara Liyanage, Jennifer Gunter, Jyotsna Batra, Ross Crawford, Hongxun Sang, Indira Prasadam

**Affiliations:** ^1^ Centre for Biomedical Technologies Queensland University of Technology Brisbane Australia; ^2^ School of Mechanical, Medical & Process Engineering Queensland University of Technology Brisbane Australia; ^3^ Department of Orthopaedics Shenzhen Hospital of Southern Medical University Shenzhen China; ^4^ Cancer Single Cell Genomics Laboratory Translational Breast Cancer Program, Olivia Newton‐John Cancer Research Institute Heidelberg Victoria Australia; ^5^ Australian Prostate Cancer Research Centre‐Queensland, Centre for Genomics and Personalised Health, School of Biomedical Science Queensland University of Technology, Translational Research Institute Brisbane Queensland Australia; ^6^ Orthopaedic Department The Prince Charles Hospital Brisbane Australia

**Keywords:** ageing, bone marrow mesenchymal stem cells, cartilage, extracellular vesicles, osteoarthritis, senescence

## Abstract

Osteoarthritis (OA), a joint disease, burdens global healthcare due to aging and obesity. Recent studies show that extracellular vesicles (EVs) from bone marrow‐derived mesenchymal stem cells (BMSCs) contribute to joint homeostasis and OA management. However, the impact of donor age on BMSC‐derived EV efficacy remains underexplored. In this study, we investigated EV efficacy from young BMSCs (2‐month‐old) in mitigating OA, contrasting them with EVs from aged BMSCs (27‐month‐old). The study used destabilisation of the medial meniscus (DMM) surgery on mouse knee joints to induce accelerated OA. Cartilage degeneration markers and senescence markers' expression levels were investigated in response to EV treatment. The therapeutic impact of EVs on chondrocytes under inflammatory responses was also evaluated. Despite having similar morphologies, EVs from young BMSCs markedly decreased senescence and improved chondroprotection by activating the PTEN pathway while simultaneously suppressing the upregulation of the PI3K/AKT pathways, proving to be more effective than those from older BMSCs in vitro. Furthermore, intraperitoneal injections of EVs from young donors significantly mitigated OA progression by preserving cartilage and reducing synovitis in a surgical OA model using DMM in mice. These findings highlight that donor age as a critical determinant in the therapeutic potential of BMSC‐derived EVs for clinical use in OA treatment.

## Graphical abstract

Extracellular vesicles (EVs) from young and older donors share phenotypic similarities, but age significantly impacts their therapeutic effects in osteoarthritis (OA) management. EVs from younger donors not only display superior chondroprotective capabilities and more effectively mitigate cellular senescence in vitro, but also enhance in vivo efficacy by slowing OA progression and reducing synovitis in a mouse model.

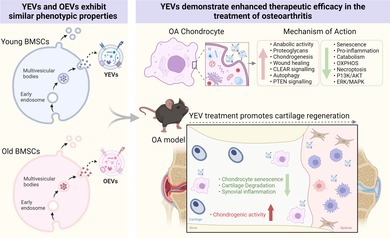

## 1 Introduction

Osteoarthritis (OA) is a prevalent age‐related musculoskeletal disorder characterised by the progressive degeneration of articular cartilage, leading to debilitating pain and functional impairment. As global life expectancy and obesity incidence continue to rise, the prevalence of OA escalates and substantially burdens healthcare systems and the quality of life in patients [1, 2]. Traditional OA management primarily aims at symptom relief through non‐pharmacological and pharmacological interventions. However, finding therapeutic strategies that can effectively slow the progression of OA and thoroughly maintain joint homeostasis continues to be a significant challenge [3, 4].

Bone marrow‐derived mesenchymal stem cells (BMSCs), owing to their unique regenerative properties, have emerged as promising candidates for innovative OA therapies [5, 6]. Beyond their inherent capacity for proliferation and directed differentiation, BMSCs exert potent immunomodulatory effects, making them ideal candidates for mitigating the pro‐inflammatory milieu that characterises OA pathogenesis [7, 8]. A growing body of research, including our previous study, has demonstrated the potential of BMSCs to alleviate cartilage degradation and stimulate tissue regeneration in preclinical OA models [9, 10, 11]. In support of these preclinical studies, BMSC‐based therapies have shown promise in reducing synovial inflammation, preventing cartilage degradation, and improving pain and symptoms in OA patients in clinical trials [7, 9, 12, 13]. However, the clinical application of BMSCs is not without its challenges, including concerns related to tumorigenesis, immune rejection, and their susceptibility to pro‐inflammatory microenvironments [14].

Recent studies increasingly demonstrated the importance of extracellular vesicles (EVs) secreted by BMSCs as a promising alternative therapy for OA [15]. EVs, a heterogeneous population of nano‐sized vesicles typically ranging in diameter from 30 to 150 nm, encapsulating a diverse array of bioactive components, have gained recognition for their pivotal role in intercellular communication and tissue homeostasis [16]. Rich in proteins, microRNAs (miRNAs), mitochondrial RNA (mtRNA), and noncoding RNAs (ncRNAs), EVs have demonstrated therapeutic potential in modulating cellular processes and orchestrating regenerative responses across a spectrum of diseases, including OA [17, 18, 19].

The effectiveness of EVs as therapeutic agents hinges on the donor cell source and their inherent characteristics [20]. Studies on EVs often present inconsistent data regarding the age and sex of participants within the cohorts, which is crucial for facilitating comparisons between studies. With projected demographic shifts favouring an increasingly ageing population, it becomes necessary to incorporate if age is a covariate in EV research. A recent study exemplified the potential of utilising EVs from young MSCs to extend lifespan in rodents, suggesting their viability as anti‐ageing therapy [21]. Nonetheless, somewhat in contrast, evidence suggests that the senescence phenotype correlates with an upsurge in total EV secretion, though the functional disparities of these vesicles remain to be elucidated. Therefore, understanding the influence of ageing on EV efficacy in regeneration will not only enhance our comprehension of the fundamental biology of ageing but also refine the design and implementation of EV‐based therapeutics in clinical conditions such as OA [22, 23].

The aim of this study is to bridge this knowledge gap by performing a comparative analysis of the therapeutic potential of EVs derived from old and young BMSCs in the pathogenesis of OA. Through a comprehensive investigation that combines in vitro and in vivo models, we seek to discern the unique contributions of age‐related factors in shaping EV functionality and their impact on cartilage microenvironment modulation. By exploring these intricacies, this study will pave the way for more effective and personalised regenerative therapies tailored to the specific age‐related characteristics of BMSC‐derived EVs.

## 2 Material and Methods

### 2.1 Isolation and Culture of Primary Mouse BMSCs

C57BL/6J mice of 2‐month‐old (“young”) and 27‐month‐old (“aged”) were used for the study. Aged mouse models were developed at Queensland Brain Institute (Brisbane, Australia) and all experiments were performed in accordance with ethics approved by the University of Queensland (AE TU 2021‐5135‐6330) and (2020/AE000231) and were conducted in alignment with ARRIVE guidelines. BMSCs were isolated from each group (*n* = 3) and cultured in Dulbecco's Modified Eagle's Medium (DMEM) containing 10% fetal bovine serum (FBS), 1% penicillin and streptomycin (Thermofisher Scientific, Scoresby, VIC, Australia) following previous protocol [24]. In brief, the limbs were dissected free of soft tissues and kept in cold PBS on ice. The bone marrow was flushed out from long bone with complete media under sterile conditions using a 22‐gauge syringe and filtration through a 70‐μm cell strainer. Isolated bone marrow cells were washed by centrifugation and cultured in a complete medium in a humidified incubator at 37°C with 5% CO_2_ [25].

### 2.2 Senescence Assay

Cell senescence was assayed by measuring senescence‐associated β‐galactosidase (SA‐β‐gal) activity using a commercial kit (Catalogue No. 9860, Cell Signalling Technology, Notting Hill, VIC, Australia) according to the manufacturer's protocol. The cells were fixed in 4% paraformaldehyde (PFA) for 15 min and stained overnight with β‐gal solution (pH 6) at 37°C. The β‐gal positive cells were viewed under a light microscope (Olympus CKX53), and the positive cells were counted using ImageJ (National Institute of Health, Bethesda, BA, USA).

### 2.3 Mitochondrial Staining

Old and young BMSCs were incubated in DMEM medium (Gibco) at 37°C with Mito Tracker Deep Red (Invitrogen) 200 nM for 30 min. After sample fixation with 4% paraformaldehyde (PFA) for 15 min, 4′,6‐diamino‐2‐phenylindole (DAPI) was used to stain nuclei. Nikon A1R laser confocal microscope (Amsterdam, Netherlands) was used to capture images.

### 2.4 Seahorse Extracellular Flux Analysis

The oxygen consumption rate (OCR) was monitored using the Seahorse Analyser XFe96 (Agilent, Santa Clara, CA, USA) following our previous study [24]. In brief, 5 × 10^3^ cells per well of young and old BMSC (*n* = 3) were seeded into XFe96 cell plates in a complete medium and incubated at 37°C for 24 h. The following day, the medium was changed with 180 μL per well of XF Base Medium (Agilent), supplemented with 1 mM pyruvate, 2 mM glutamine and 10 mM glucose, and adjusted to a pH of 7.4. The Cell Mito Stress assay was run with final concentrations of 1 μM Oligomycin, 2 μM Carbonyl cyanide 4‐(trifluoromethoxy) phenylhydrazone (FCCP), and 0.5 μM each of Rotenone and antimycin A. After 15 min equilibration, OCR was recorded every 6 min (3 min mixing and 3 min measuring), with 3 basal measurements and 3 measurements after injection of each compound. Upon completion of the analysis, cells were stained with Hoechst 33342 at 1 μg/mL final concentration, and the cell count was measured using the InCell 2200 (GE Healthcare). Images we quantitated using a custom pipeline in Cell Profiler (Broad Institute). The results were normalised with cell number per corresponding well.

### 2.5 Isolation of BMSC‐EVs

The isolation procedures of young and aged murine BMSCs were performed based on the previous study [24, 26]. In detail, after cells reached 70%–80% confluence, the complete medium was changed by DMEM medium with 10% EV‐depleted FBS and 1% penicillin and streptomycin. The conditioned medium was collected after 48 h incubation. This medium was centrifuged sequentially (Beckman Coulter Microfuge 18 Centrifuge) at 300 × g at 4°C for 10 min to eliminate detached cells. The supernatant was subsequently filtered through 0.22 μm filters to eliminate apoptotic bodies and cell debris. Then, the supernatant was ultracentrifuged at 100,000 × g at 4°C for 90 min using a Beckman Coulter OptimaTM L‐90K Ultracentrifuge equipped with a SW41 rotor to sediment EVs. Afterwards, the precipitate was re‐suspended in ice‐cold PBS and subjected to another round of centrifugation at 100,000 × g at 4°C for 90 min using a Beckman Coulter OptimaTM MAX‐80XP with a TLA 110 rotor. The EV pellet was resuspended in sterile PBS, aliquoted into multiple vials to minimise the impact of freeze‐thaw cycle  and stored at −80°C for subsequent experiments. EVs isolated from the conditioned medium of young BMSCs were termed YEVs, whereas those from aged (old) BMSCs were referred to as OEVs.

### 2.6 Nanoparticle Tracking Analysis (NTA) and Size Distribution

To assess the absolute size distribution of BMSC‐EVs, YEVs and OEVs were analysed using NTA with an NS300 NanoSight instrument (ATA Scientific), and automatic settings were applied for maximum jump distance and blur accordingly. 20 μL of each EV sample was diluted in 500 μL of filtered PBS and then assessed. The particles were tracked and sized using NTA, which relies on Brownian motion and the diffusion coefficient. Data analysis was performed using the NTA software 3.0 (ATA Scientific). For each biological replicate, measurements were taken in triplication and averaged.

### 2.7 Atomic Force Microscopy (AFM)

Freshly cleaved mica sheets were treated with NiCl_2_ and then rinsed with deionised water. After treatment, 10 μL of EV suspension was gently added to the mica sheets. The samples were stored overnight at 4°C. The following day, they were rinsed with deionised water and air‐dried within a laminar flow hood. NanoWizard II atomic force microscope (JPK, Germany) was used to confirm the presence of EVs. The examination was performed in tapping mode by cantilevers. Image acquisition was conducted at a scan rate of 0.25 Hz [27]. Software from JPK was utilised for image processing and subsequent analysis.

### 2.8 Transmission Electron Microscopy (TEM) and Morphological Analyses

Morphological assessments of EVs were performed using TEM. A 5 μL droplet of the vesicle suspension was placed onto copper TEM grids coated with carbon/formvar (Emgrid Australia, Adelaide, Australia). Afterwards, the grids were stained with a 1% solution of uranyl acetate for approximately 1 min and then allowed to dry prior to transmission electron microscopy (JEM‐1400, JOEL, Tokyo, Japan), acquiring the images [24]. For cryogenic TEM imaging, EV samples were prepared with Leica EM GP2 robotic vitrification system under controlled conditions (22°C and 95% relative humidity). A 2 μL EV suspension was applied on a carbon‐coated perforated formvar film on a 300‐mesh copper TEM grid. Excess solution was automatically blotted for 3 s before rapidly plunging into liquid ethane at approximately −183°C. Prior to this, the grids were treated with ethyl acetate and subjected to gentle glow discharge with plasma to enhance surface charge and improve sample distribution. The prepared grids were stored in liquid nitrogen until imaging. Imaging was performed using a Jeol Cryo ARM 200 (JEM‐Z200FSC) TEM in a frozen hydrated state at −176°C. The microscope was equipped with a cold field emission gun (FEG) and an in‐column Omega energy filter. Images were captured with zero energy loss at an acceleration voltage of 200 kV and a filter setting of 20 eV. Furthermore, the images were recorded under low‐dose conditions using the SerialEM software (Mastronarde, 2005) and a Gatan K2 direct detector camera to minimise exposure [28].

### 2.9 Western Blots

To identify the EV markers, a western blot was performed in accordance with the protocol that had been previously described by our group [24, 29]. Briefly, samples were lysed in radioimmunoprecipitation assay (RIPA) buffer and protein concentrations were determined with a Pierce BCA Protein Assay Kit (Thermofisher Scientific, Scoresby, VIC, Australia) according to the manufacturer’s instructions. Lysates were incubated with reducing sample buffer for electrophoresis. Following separation by 10% SDS‐PAGE, the samples were then transferred to polyvinylidene fluoride membranes (Millipore). 5% BSA in PBS‐T (0.5% Tween‐20) was used to block the membrane and then incubated with the primary antibodies at 1:1000 overnight: Alix (Catalogue No. 92880) and TSG101 (Catalogue No. 72312S) from New England Biolabs. Odyssey Infrared Imaging System (LI‐COR Biotechnology, USA) was used to detect the protein bands.

### 2.10 Human Tissue Collection and Cell Isolation

Femur cartilage was obtained from three patients age between 60‐74 who were undergoing primary total knee arthroplasty as described previously [30]. The Queensland University of Technology and the St Vincent Private Hospital Ethics Committees approved this research with respect to human ethics, and all participants provided informed consent (ethics number: #1400001024). Cartilage was collected from the region of the femoral condyle, identified as intact and healthy, with a Kellgren‐Lawrence (KL) grade of 1. Cartilage was finely chopped and rinsed three to four times with PBS. and then digested with a 1% type II collagenase solution (Invitrogen, Lakewood, NJ) to isolate primary chondrocytes, as previously described [30]. After centrifugation, the cells were cultured in a low‐glucose DMEM medium with 10% FBS and 1% PS.

### 2.11 In Vitro PHK26 Tracking for BMSC‐EVs

EVs were labelled for co‐localisation studies using the PKH26 Red Fluorescent Cell Linker Kit (Sigma‐Aldrich, Australia) following the manufacturer's guidelines. Briefly, the isolated YEV and OEV were diluted using Diluent C from PKH26 kits and incubated with PHK26 dye for 5 min at room temperature. The reaction was stopped with a DMEM medium containing 1% EV‐free FBS, and extra dye was removed by ultracentrifugation. The labelled EV pellets were resuspended in PBS and cocultured with chondrocytes at 37°C for 24 h. Following the incubation, 4% PFA was used to fix the cells and DAPI staining was performed. Fixed samples were mounted on a glass slide with mowiol (Sigma‐Aldrich, Sydney, Australia), and images were captured using a Nikon A1R Confocal laser scanning microscope (Amsterdam, The Netherlands).

### 2.12 Induction of OA‐Like Senescence in Human Chondrocytes and Treatment With BMSC‐EVs

Freshly isolated human chondrocytes were seeded in 24‐well plates with complete DMEM medium and cultured at 37°C with 5% CO_2_; the cells were induced with or without 10 ng/mL of IL‐1β (Preprotech, Thermofisher, Australia) for 24 h. Subsequently, the OA chondrocytes were treated with 10 μg/mL of YEV or OEV for 24 h. Following EV treatment, Calcein‐AM and Propidium Iodide (PI) were used to stain chondrocytes (Thermofisher, Australia) for 10 min at 37°C with 5% CO_2_ to detect the viable and dead cells, respectively. Cells were then imaged using an Eclipse TE2000‐U fluorescent microscopy (Nikon, Japan).

### 2.13 Cell Proliferation Assay

Proliferation of cells was determined using a conventional MTT [(3‐(4,5‐Dimethylthiazol‐2‐yl)‐2,5‐Diphenyltetrazolium Bromide)] (Sigma‐Aldrich, Sydney, Australia) substrate as described with modifications. Fresh isolated young and old BMSCs were cultured at a density of 5 × 10^3^ per well with the complete medium in triplicate in a 96‐well plate and incubated for 24, 48 and 72 h, respectively. To evaluate the effect of YEV and OEV on cell proliferation, human P1 chondrocytes were cultured in 96‐well plates in triplicate, with each well containing 5 × 10^3^ cells and treated with YEV or OEV (5, 10, 20 μg/mL) respectively or an equal volume of DMEM medium (as control) for 24 h. Post‐treatment, 10 μL of 5 mg/mL MTT solution was added to each well. The plate was incubated for 4 h at 37°C with 5% CO_2_. Following incubation, the medium‐MTT mix was then replaced with 100 μL of dimethyl sulfoxide, which helps to solubilise the formazan crystals, and absorbance was measured at 570 nm Benchmark Plus microplate spectrophotometer (Tacoma, WA, USA).

### 2.14 Bovine Explant and BMSC‐EVs Treatment Ex Vivo

Fresh adult bovine knee joints were obtained from a local abattoir. Bovine cartilage explants from the femoral condyles of young bovine knee joints (3–5 months old) were harvested using a 4.0 mm biopsy punch. The full‐thickness articular cartilage explants were cultured in a complete medium at 37 °C with 5% CO_2_ for 24 h. To study the therapeutic effects of YEV or OEV, these explants were treated with 10 ng/mL IL‐1β (Preprotech, Thermofisher, Australia) with or without 10 μg/mL of YEVs or OEVs. After 24 h, the media were collected and stored at −80°C to quantify sulphated glycosaminoglycans (sGAG) using a colorimetric dimethyl methylene blue assay (DMMB) following the manufacturer's protocol. The treated bovine cartilage explants were fixed using 4% PFA, and embedded in paraffin, sectioned and stained using the Safranin O staining according to our previous protocol [26].

### 2.15 RNA Extraction and Quantitative Real‐Time PCR

Using TRIzol reagent (ThermoFisher, 15596018), total RNA from the cells was isolated following the previously described protocol. The SensiFAST cDNA Synthesis Kit (Catalogue No. BIO‐65053, Bioline) was used to perform the reverse transcription. SYBR green quantitative real‐time polymerase chain reaction (PCR) SuperMix Plus (Catalogue No. 4312704, Thermofisher) was used to detect gene transcript through the QuantStudio Real‐Time PCR system (Applied Biosystems, Thermo Scientific, VIC, Australia). The (2 − ΔΔCt) method was employed for relative quantification, with β‐actin/GAPDH used as internal controls [24]. The primer sequences are provided in Table S1.

### 2.16 Sample Preparation and Proteomics Analysis

Following EV treatment, C28/I2 human chondrocyte cell line (gift from Prof Yin Xiao, Griffith University, Gold Coast, Australia) were lysed and solubilised by vortexing and 1‐h incubation with gentle agitation in 2× SDS lysis buffer containing 20 mM dithiothreitol (DTT) at 70°C. The extracts were alkylated using 40 mM iodoacetamide in the dark at room temperature for 30 min, followed by sonication and clarification via centrifugation at 13,000 g for 8 min. The extracts were acidified with 12% phosphoric acid and precipitated with nine volumes of S‐Trap binding buffer (90% MeOH, 100 mM final Tris). Overnight digestion at 37°C with 1 μg of trypsin was conducted on a shaker. Digested peptides were eluted sequentially with 5%, 50% and 75% acetonitrile solutions in 0.1% formic acid, concentrated using a dry vacuum centrifuge and resuspended in 5% acetonitrile with 0.1% formic acid and resuspended in 20 μL buffer. Peptides were separated using an Ultimate 3000 RSLC ultra‐high‐performance liquid chromatography (UHPLC) system (Thermo Fisher Scientific, Waltham, Massachusetts, USA), and mass spectrometry was performed with an Exploris 480 mass spectrometer (Thermo Fisher Scientific, Waltham, Massachusetts, USA).

### 2.17 LC–MS/MS Data Analysis

Protein abundance/intensity values from all replicates were analysed using the MetaboAnalyst 5.0 online platform, adhering to the protocol previously published [9]. Principal component analysis (PCA) was applied to the first three principal components of log2‐transformed normalised areas. Heat maps were generated with normalised protein intensities and clustered using the Ward algorithm. Hierarchical clustering based on Euclidean distance organised the row and column order. Fold changes in protein expression between treatment groups were calculated using the log2 transformation method. Differentially expressed proteins were identified with thresholds set at ±Log2FC ≥ 0.5 and *p* ≤ 0.05 and further analysed in Ingenuity Pathway Analysis (IPA) to identify significant changes in canonical signalling pathways and upstream regulators, with Z‐score thresholds of ±1.50 and *p* ≤ 0.05 indicating significant dysregulation.

### 2.18 Establishment of Mice OA Model and EV Treatment

The study was conducted following approval by the Institutional Animal Care and Use Committee and Institutional Biosafety Committee of Central–South University (2022‐0165). Twenty‐four male C57BL/6 mice, aged 7 weeks, were purchased from the Animal Centre of Central–South University (Changsha Shi, Hunan Sheng, China). Animals were randomised into three groups of six animals each. OA was induced by opening the right knee using a medial parapatellar approach, and the medial meniscal ligament was dissected to destabilise the medial meniscus. The sham surgeries were performed similarly, except for the medial meniscal ligament, and the meniscus was not disturbed. Four weeks post‐surgery, mice that had undergone DMM surgery were randomly divided into 3 groups. Each group received intraperitoneal injections as follows: 50 μg YEV in 150 μL PBS, 50 μg OEV in 150 μL PBS, or 150 μL PBS on days 1, 3, 5, 7 and 10 after surgery. Intraperitoneal injections of 150 μL PBS were given to the mice that had undergone the sham surgery. All mice were euthanised, and the knee joints were taken for additional investigation 8 weeks after the surgery.

### 2.19 Histological Evaluation of Knee Joints

Knee joint samples were fixed in 4% PFA for 24 h, followed by decalcification with 10% ethylenediaminetetraacetic acid (EDTA). The decalcified samples underwent dehydration and paraffin embedding and were then sectioned at a thickness of 5 μm. In accordance with the previous protocol, Safranin O/Fast Green staining was performed for the sections. Two independent assessors then assessed the sections for synovial inflammation as described previously [31] and cartilage degradation. The synovitis was scored using a standardized scoring system based on histopathological changes observed in the synovial membrane, including cellularity, synovial lining thickness, and presence of inflammatory infiltrates [31]. The modified Mankin score was applied to evaluate cartilage pathological changes [26]. To detect the proteoglycans in bovine cartilage explants, the samples were sectioned at 5 μm thickness and stained with safranin O. Leica SCN400 slide scanner (Leica Biosystems, Australia) was used to image samples at 40× magnification.

### 2.20 Immunofluorescent Staining

Immunofluorescence staining was performed according to our previous studies [26]. Briefly, EV‐treated human chondrocytes were fixed with 4% PFA, followed by PBS wash. To block the nonspecific binding, 2% bovine serum albumin was used, and permeabilisation was done at room temperature using 0.25% Triton X‐100/PBS solution. The primary antibody against collagen II (Catalogue No. MBS851721, My BioSource; 1:200 dilution) was incubated with cells at 4°C overnight. Following incubation and PBS washes, samples were incubated with corresponding fluorescent secondary antibody (Alexa Fluor 488 Catalogue No. AB150081, Abcam; 1:500 dilution) for 1 h at room temperature. DAPI was used to stain the nuclei. Nikon A1R confocal microscope was used to observe the immunofluorescence. Fluorescence intensity was quantified from fluorescence images in each group using ImageJ (National Institute of Health, Bethesda, BA, USA).

For the evaluation of cartilage degradation and senescence in mouse knee joints, immunohistochemical methods were performed accordingly [26, 29]. Slides were incubated with a primary antibody against p21 (Catalogue no. MBS9458822, My BioSource; 1:100 dilution), ACAN (Catalogue No. MA3‐16888, Thermofisher; 1:100 dilution), and ADAMTS5 (MBS2520611, My BioSource, 1:50 dilution). Following overnight incubation at 4°C, the sections were further incubated with the HRP polymer secondary antibody, and the visualisation of antibody complexes was accomplished using a diaminobenzidine (DAB) substrate from En Vision kit (Catalogue no. k5007, Dako, Australia). Mayer's haematoxylin was utilised for counterstaining. Comprehensive imaging of the entire knee joint was conducted using a Leica SCN400 slide scanner (Leica Biosystems, Australia) to assist histomorphometry measurements. Image analysis was executed utilising Image J (National Institute of Health, Bethesda, BA, USA). The total cells and the number of cells displaying immuno‐positive staining were counted in three distinct fields of the tibial plateau superior within three consecutive sections per specimen for each group. These counts were then normalised to represent the number of cells per 100 total cells.

### 2.21 Statistical Analysis

All graphical representations and statistical analyses were performed using GraphPad Prism (version 9.0, GraphPad, USA). All experimental data were reported as mean ± standard deviation (SD). Statistical differences between the two groups were assessed using Student's *t*‐test, while one‐way analysis of variance (ANOVA) was utilised for comparisons involving more than two groups. For multiple comparisons, the Brown‐Forsythe test was applied. The sample sizes for each experimental group are detailed in the figure legends. A *p*‐value of less than 0.05 was considered to indicate statistical significance.

## 3 Results

### 3.1 Increased Cartilage Degeneration and Accumulation of Senescence Phenotype in Older C57BL/6J Mice

**FIGURE 1:**
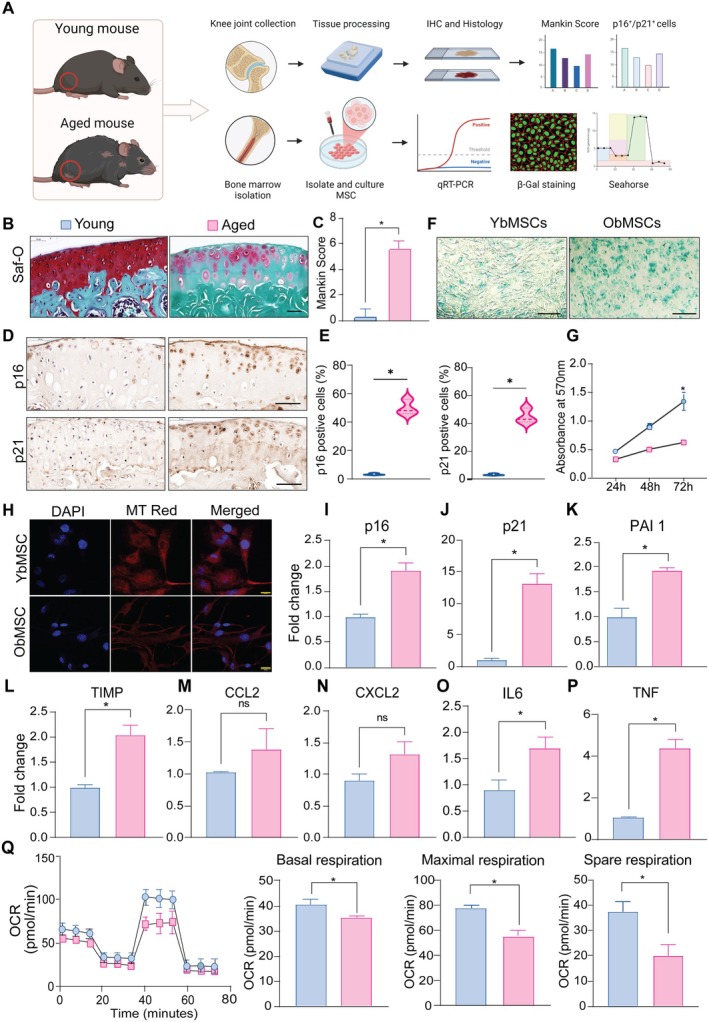
Characterization of cartilage and BMSCs isolated from young and aged mice. (A) Schematic representation of experimental design. (B) Representative images of safranin O/fast green staining of knee joints of young and aged mice. Scale bar, 50 μm. (C) Quantification of Mankin scoring of Knee joints (D) Immunostaining for p16 (upper) and p21 (lower). Scale bars, 50 μm. (E) p16 and p21 immunostaining quantitative analysis of articular cartilage from aged and young mice (F) Senescence‐associated‐β‐galactosidase staining of BMSCs isolated from aged and young mice. Scale bar 100 μm. (G) MTT cell proliferation assay of young and old BMSCs (H) Mito‐tracker red staining analysis of young and old BMSCs. Scale bar 20 μm. (I to P) qRT‐Cs. (Q) Oxygen consumption rate of young and old BMSsC. Data are presented as mean ± SD from three independent experiments ( *p ≤* 0.05; ns: not significant).

We first aimed to assess whether natural ageing shows any changes in cartilage and BMSCs. For this purpose, we collected the knee joints from young (2‐month‐old) mice and aged (27‐month‐old) mice (Figure 1A). To explore the age‐related changes occurring in the knee joint, we performed Safranin O/Fast Green staining. Histological examination of knee joints from both aged and young mice revealed an age‐related decline in safranin O staining of proteoglycans and the presence of cartilage surface irregularities. Mankin Scores showed cartilage degradation in the aged mice vs. young mice (Figure 1B,C). Next, we tested whether senescence develops after articular cartilage degeneration in mice. Expression of senescence‐associated markers p16INK4a and p21 in articular cartilage was characterised by immunohistochemistry. Aged mice, when compared to young mice, had a greater number of p16INK4a and p21‐expressing chondrocytes in the articular cartilage (Figure 1D,E). To investigate whether senescence affects the multilineage differentiation potentials of BMSCs, osteogeneic, adipogenic and chondrogenic differentiation of young and old BMSCs were assessed on 14 days. As shown in Supplementary Figure 1, both groups exhibited mineralization and lipid deposit accumulation, as well as the presence of proteoglycans. We used SA‐β‐gal staining and a cell proliferation assay to evaluate the impact of ageing on BMSCs. As demonstrated in Figure 1F, the number of SA‐β‐gal+ cells significantly increased in old BMSCs compared to the young BMSCs. The proliferation rate in the older BMSCs significantly decreased compared to the younger BMSCs (Figure 1G). Mitochondrial dysfunction causes disturbances in reactive oxygen species metabolism, leading to DNA impairment and the activation of the DNA damage response, thereby accelerating the ageing process. MitoTracker Deep Red staining showed that mitochondria staining was obviously decreased in the old BMSCs compared to those in the young cells (Figure 1H).

We evaluated the transcript level of senescence‐associated genes. Compared to BMSCs obtained from young mice, the BMSCs from aged mice showed higher expression of a group of senescence genes (Figure 1I–P). To further elucidate the functional alterations in the cellular metabolic activity of BMSCs, we assessed the OCR in both young and old BMSCs, as depicted in Figure 1Q. After the addition of FCCP, we observed that the maximal respiration of old BMSCs was considerably lower than that of young BMSCs. The spare respiratory capacity of old BMSCs was significantly lower than that of young BMSCs when FCCP was introduced. This indicates a substantial decrease in OCR) in old BMSCs compared to young BMSCs, highlighting impaired oxidative phosphorylation in the old BMSCs. These results collectively indicate increased senescence in old BMSCs and impaired metabolic activity.

### 3.2 EVs Isolated From Young and Old BMSC Exhibit Similar Phenotypic Properties

**FIGURE 2:**
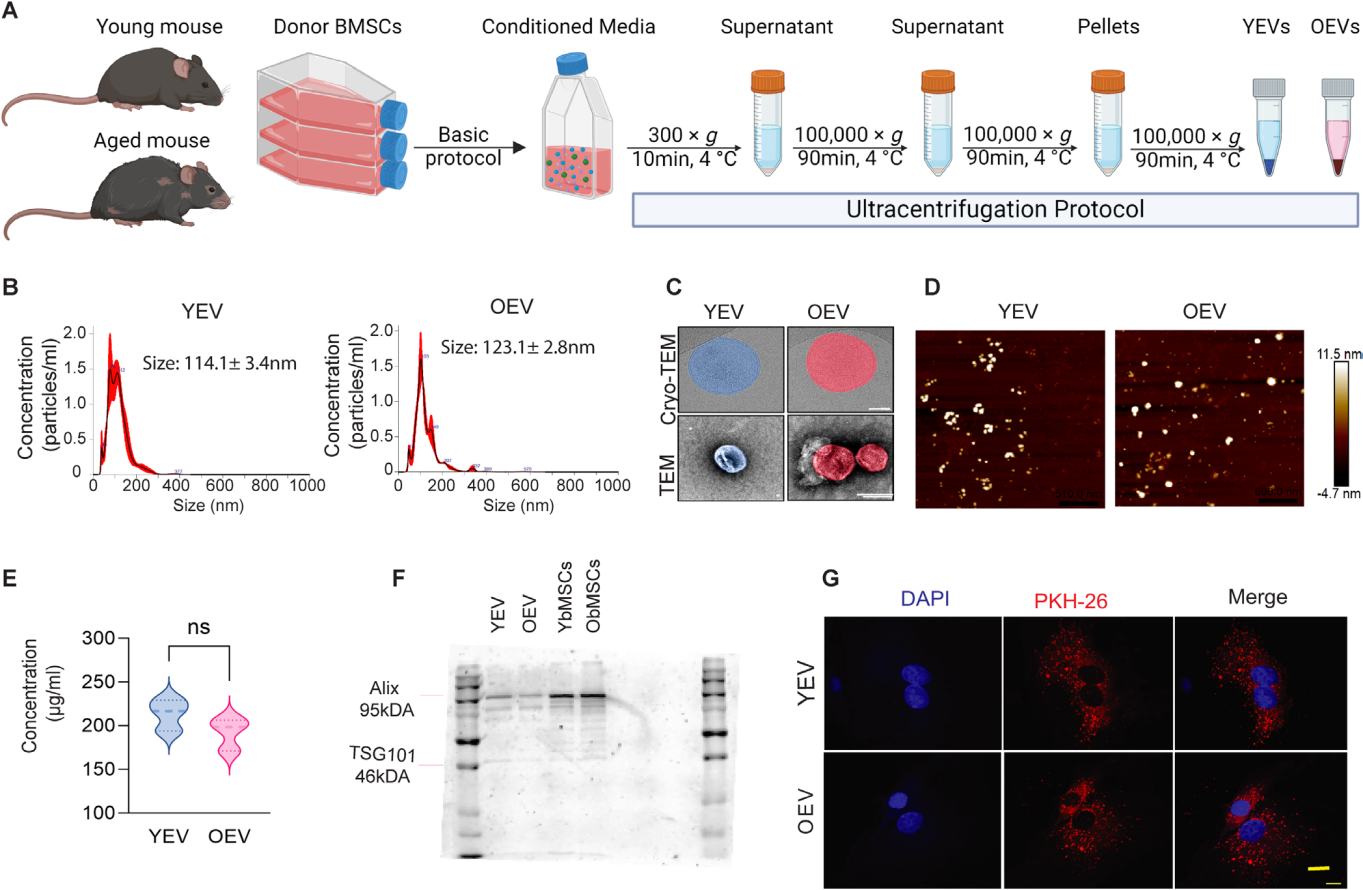
Old and young BMSC‐derived EVs showed similar characteristics. (A) Schematic diagram of the EVs isolation procedure from young and old BMSCs (B) Representative nanoparticle tracking analysis of young and old EVs size distribution (C) Cryo‐TEM (upper) images demonstrate the double‐membrane structure of YEV and OEV. Scale bar, 10 nm and transmission electron microscopy (lower) image shows the cup‐shaped structure of EVs. Scale bar, 100 nm. (D) The three‐dimensional topography images of EVs obtained using atomic force microscopy characterisation (E) Protein concentration of YEVs and OEVs (F) Western blot analysis for EV protein markers (Alix and TSG 101) in young BMSC lysate, YEVs, old BMSC lysate and OEVs. (G) Uptake of YEVs and OEVs by primary human chondrocytes were observed under a fluorescent microscope after 24 h of coculture. EVs were stained with PKH26 (red), and DAPI (blue) was used to stain chondrocyte nuclei; scale bar, 20 μm. Data are presented as mean ± SD from three biological replicates for imaging and technical replicates for quantitative analyses (* *p* ≤ 0.05; ns: not significant).

Old and young BMSCs were cultured in EV depleted medium, and the condition medium was collected after 48 h. Figure 2A shows the schematic representation of EV isolation by centrifugation. The characterisation of YEV and OEVs was performed following the guidelines outlined by the minimal information for studies of extracellular vesicles (MISEV) recommendations [32]. The EVs were characterised using NTA, which showed that the size of the EVs ranged within an approximate range of 114.1 ± 3.4 nm for YEV and 123.1 ± 2.8 nm for OEVs, corresponding to the standard size of EV (Figure 2B). Continuing with the characterisation, we performed cryo‐transmission electron microscopy and transmission electron microscopy to observe the morphology of EVs. Cryo‐EM revealed that YEV and OEVs had a similar double membrane structure and spherical shape morphology. TEM also showed similar morphology for both the EVs (Figure 2C). Furthermore, the physical attributes and three‐dimensional structure of the EVs were investigated through atomic force microscopy (AFM) (Figure 2D). The mean protein concentration for both the EVs was approximately 300 μg/mL and statistical analysis did not reveal any significant difference (Figure 2E). Both YEV and OEV showed the expression of surface marker proteins (Alix and TSG101) in western blots (Figure 2F). Finally, to track EVs and their uptake by the normal chondrocytes, we labelled OEVs and YEVs using PKH26. Figure 2G shows red fluorescence localised in the cytoplasm of chondrocytes, suggesting that both OEVs and YEVs were uptake by normal chondrocytes. These results indicate that EVs were successfully isolated from the conditioned medium; however, no significant phenotypic difference exists between OEVs and YEVs despite their parent cells showing age‐related changes.

### 3.3 YEVs Reduced the OA‐Associated Chondrocyte Senescence

**FIGURE 3:**
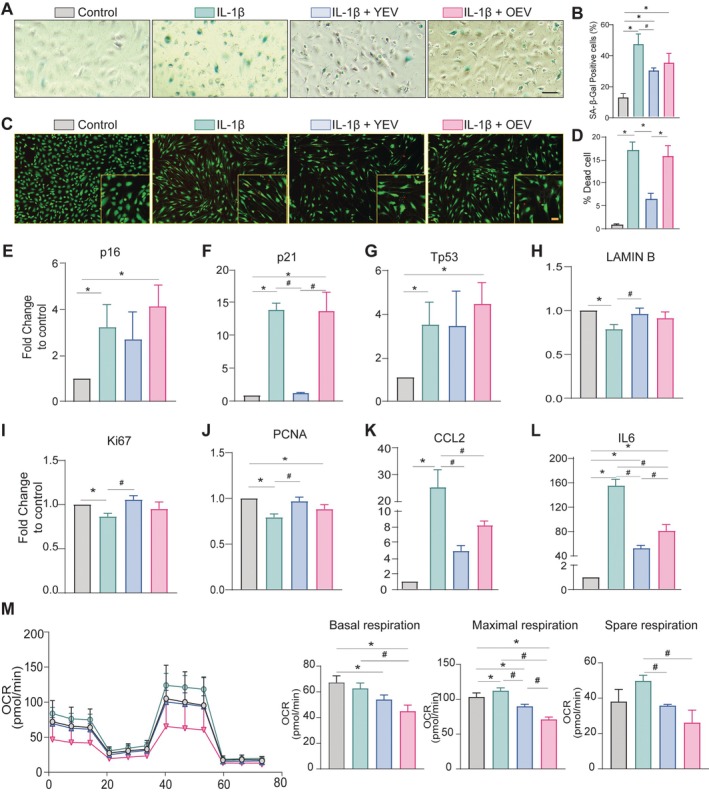
Young BMSC‐derived EVs reduce senescence in vitro. (A) SA‐β‐gal staining of chondrocytes induced with IL‐1β and treated with young and old EVs (n = 3). Scale bar, 100µm. (B) Quantification of SA‐β‐gal positive cell (% area) * *p* ≤ 0.05. (C) The effect of IL‐1β, YEVs and OEVs on the chondrocyte viability was detected by the cell live/death experiment; green represents live cells, while red represents dead cells. Scale bar, 20µm. (D) Quantification of the dead cell as a percentage. * *p* ≤ 0.05. (E‐L) The gene expression of senescence and SASP markers (p16, p21, p53, TIMP, IL‐6 and CCL2) in IL‐1β‐induced chondrocytes and IL‐1β‐induced chondrocytes treated EVs. (M) Seahorse mitochondrial stress test analysis of IL‐1β‐induced chondrocytes cocultured with young and old EVs and negative control. Data are presented as mean ± SD from three independent experiments (*compared to control, *p* ≤ 0.05; #compared to control, *p* ≤ 0.05).

SA‐β‐gal Cellular senescence is a significant contributor to the pathogenesis of OA. Human chondrocytes can develop senescence through different mechanisms, including replicative and stress‐induced premature senescence [33]. Firstly, the influence of YEVs and OEVs on proliferation capacity of chondrocytes were determined. Chondrocytes treated with YEVs exhibited a significant increase in proliferation in a dose‐dependent manner compared to the non‐treated group. OEVs also strongly supported cell proliferation, but no differences were observed among various doses. A concentration of 10 µg/mL of EVs was the minimum effective dose that did not affect cell growth differently between chondrocytes treated with YEVs and OEVs (Supplementary Figure 2). Increased expression of cell cycle arrest‐related genes such as p16, p21 and p53 induces senescence directly. The growth arrest, including other factors, leads to the production of bioactive senescence‐associated secretory phenotypes (SASP) like TIMP‐1 and CCL2, which are induced due to these morphological changes [34]. Next, we examined the effect of YEVs and OEVs on IL‐1β‐stimulated chondrocytes using SA‐β‐galstaining, live/dead assay, and RT‐qPCR. We found that YEV‐treated cells exhibited a decrease in senescence and a reduction in the percentage of dead cells compared to the IL‐1β group; however, OEV treatment did not positively affect the IL‐1β‐stimulated chondrocytes (*p* ≤ 0.05) (Figure 3A–D). Furthermore, as shown in Figure 3E–G, the expression of p16, p21 and p53 was significantly upregulated in IL‐1β treated chondrocytes compared with the control chondrocytes. The transcript levels of all these senescence genes were significantly decreased in IL‐1β‐treated cells when treated with YEVs. Conversely, while OEVs did not reduce the expression of the p16, p21 and p53 genes. Another senescence maker, LAMINB expression, was decreased in IL‐1β‐treated cells; however, YEVs significantly upregulated its expression. The proliferation markers Ki67 and PCNA expression decreased in IL‐1β‐treated chondrocytes but increased substantially after YEV and OEV treatment (Figure 3I,J). The proinflammatory markers IL6 and CCL2 were upregulated significantly in IL‐1β‐treated cells. However, in the presence of YEVs, the expression level of these markers decreased substantially, while OEV treatment was not as effective as YEVs (Figure 3K,L). Using a mitochondrial stress test, real‐time changes in the cellular oxygen consumption rate (OCR) were obtained for all four groups by a Seahorse flux analyser. The basal, maximal and spare respiration were reduced in IL‐1β treated chondrocytes compared to the control chondrocyte group; however, the OCR levels in chondrocytes treated with YEVs were significantly enhanced while OEV treated chondrocytes showed a decrease in OCR levels (Figure 3M). Altogether, this suggests that YEVs showed better potential than OEVs to suppress the chondrocyte senescence phenotype.

### 3.4 YEVs and OEVs Exhibit Differential Chondroprotective Effects

**FIGURE 4:**
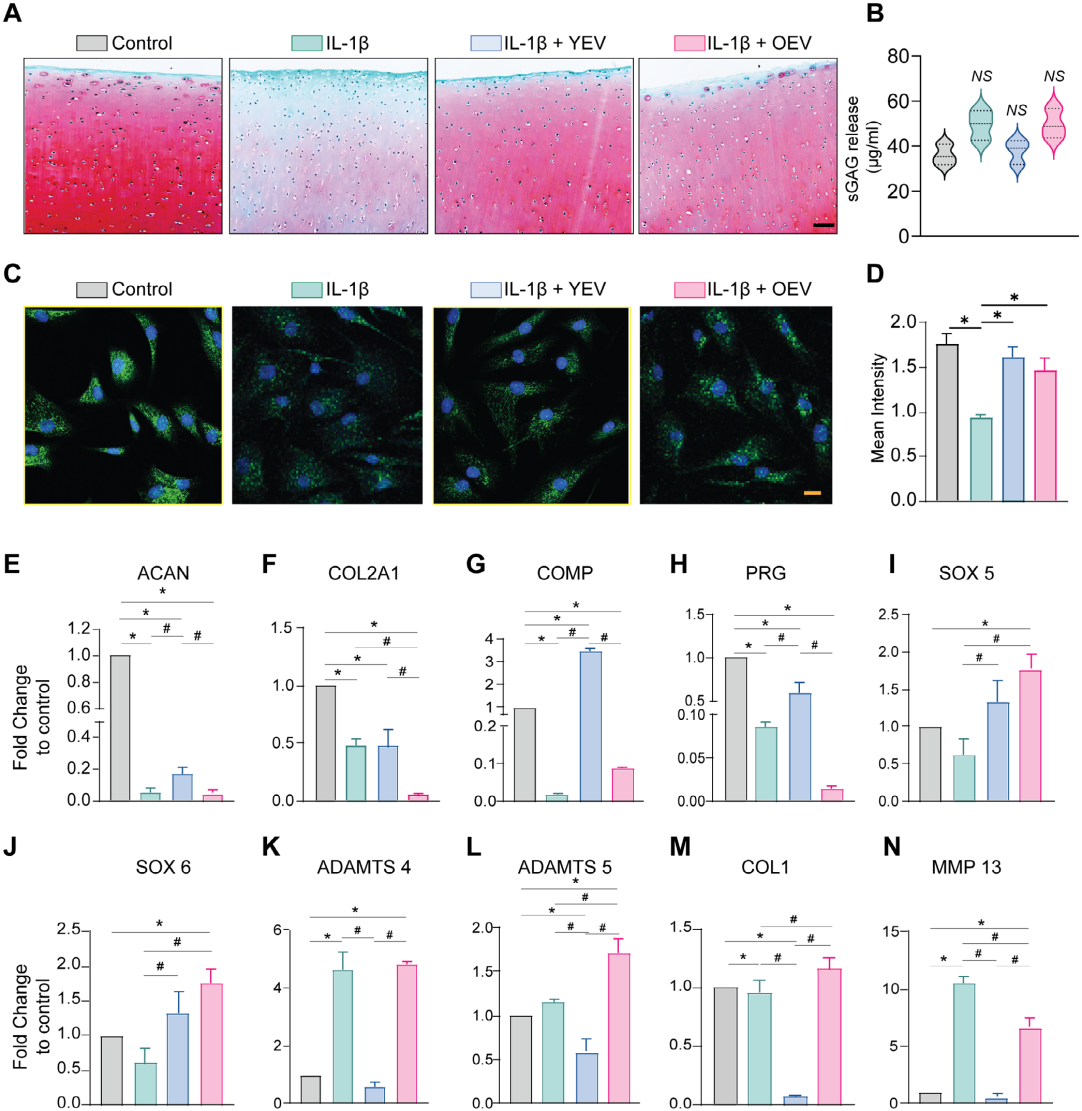
EVs from young BMSCs show superior chondroprotective effects than OEVs in an ex vivo and in vitro model. (A) Representative images of Safranin O/Fast green staining of IL‐1β‐induced bovine cartilage cocultured with young and old EVs and negative control. Scale bar, 100µm. (B) Extracellular GAG release of bovine cartilage explants cocultured with young and old EVs and negative control. (C) Immunofluorescence staining for collagen II in IL‐1β‐induced chondrocytes treated with EVs. Scale bar, 20µm. (D) Mean intensity of collagen II positive cells with different treatments. (E‐N) qRT‐PCR analysis of IL‐1β‐induced chondrocytes cocultured with young and old EVs and negative control. Data represented as mean ± SD (n = 3) for both imaging and quantitative analyses; *compared to control, *p* ≤ 0.05; #compared to IL‐1β, *p* ≤ 0 .05; ns: not significant.

To examine whether EVs from young and old BMSCs exert chondroprotective effects on OA progression in a 3D environment, we employed IL‐1β‐stimulated bovine articular cartilage explants, a well‐established ex vivo model for OA. Safranin O staining decreased significantly in bovine articular cartilage stimulated ex vivo with IL‐1β and showed an increase in the concentration of extracellular sulphated glycosaminoglycan (Figure 4A,B). Notably, YEVs, but not OEVs, prevented cartilage degradation caused by IL‐1β, resulting in similar levels of Safranin O content and sGAG quantity in the culture media as the control group (Figure 4A,B). To further assess the therapeutic effects of YEVs and OEVs in vitro, human chondrocytes were treated with IL‐1β to induce an OA‐like phenotype. They were subsequently subjected to treatments with YEVs and OEVs, respectively. The data from immunofluorescence showed that IL‐1β significantly depleted the expression of Col II; however, this effect was reversed by YEV and OEV treatment (Figure 4C,D). To better understand how YEVs and OEVs protect chondrocytes, the gene expression of catabolic and anabolic markers was assessed by qRT‐PCR. IL‐1β induction resulted in an OA‐like phenotype in chondrocytes, characterised by a significant reduction in the expression of anabolic gene markers, including ACAN, COL2A1, COMP, PRG, SOX5 and SOX6, and an elevation in catabolic gene markers such as ADAMTS4, ADAMTS5 and MMP13 when compared to the control group (Figure 4E–N). The catabolic effect of IL‐1β on chondrocytes was effectively reversed by YEVs and showed a significantly increased expression of anabolic factors, including ACAN, COL2A1, COMP, PRG, SOX5 and SOX6. Unlike the ex vivo findings, in vitro treatments with OEVs did not influence the gene expression of anabolic or catabolic markers under IL‐1β stimulation. Overall, these findings highlight that YEVs can improve the balance between anabolic and catabolic effects more effectively than OEVs, especially in osteoarthritic conditions.

### 3.5 EV Treatment Changes Protein Expression in Chondrocytes

**FIGURE 5:**
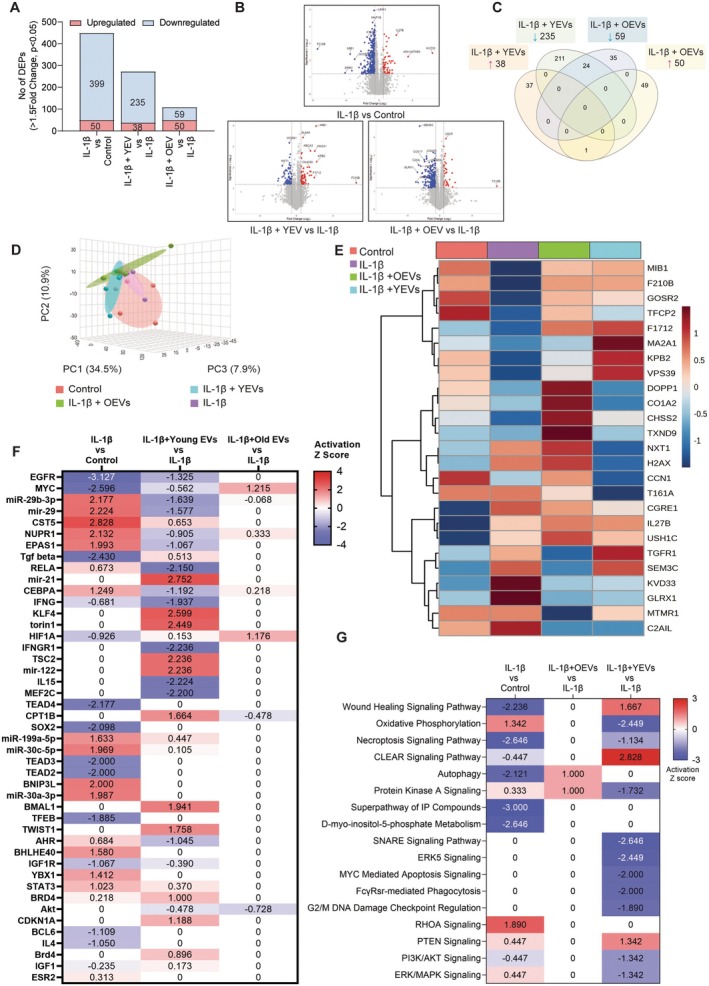
Differentially expressed proteins (DEPs) in IL‐1β induced chondrocytes and IL‐1β induced chondrocytes after YEVs and OEVs treatment. (A) Graph indicating the number of differentially expressed proteins (DEPs) upregulated and downregulated after YEV and OEV treatment (*n* = 5). (B) Volcano plots representing the DEPs based on their fold change and the significance of the change in expression in each group. (C) Venn diagram representing the overlap of proteins that are significantly upregulated and downregulated in IL‐1β induced chondrocytes and the YEV and OEV treatment. (D) The graph demonstrating the 3D principal component scores plot for the proteomic profiles of all four groups. (E) Heatmap representation of the normalised protein expression/intensity Z‐scores for the top 25 DEPs in each group (Negative control, IL‐1β, chondrocytes treated with YEV, and chondrocytes treated with OEV). (F) Summary of the top significantly dysregulated upstream regulators in each group. (G) Summary of the top canonical pathways significantly change after EV treatments. A threshold of ±Log2 FC ≥ 0.5 and *p* ≤ 0.05 was considered to identify the DEPs in each group. Activation/inhibition Z‐score threshold ±1.50 and *p* ≤ 0.05 were considered to make predictions of the activated (Red) or inhibited (Blue) canonical pathways and upstream regulators. *n* = 3 indicates three biological replicates for both imaging and quantitative analyses.

To examine the molecular mechanisms altered upon the EV treatment on IL‐1β induced chondrocytes, we profiled the proteome of chondrocytes using mass spectrometry analysis. In summary, proteomic analysis has identified 6357 proteins, of which 763 differentially expressed proteins (DEPs) were found in the three treatment comparison groups. Of note, 399 proteins were downregulated, and 50 proteins were upregulated after IL‐1β treatment, whereas following YEV treatment, 235 proteins were downregulated, and 38 proteins were upregulated. OEV treatment led to the upregulation of 50 proteins, while 59 proteins were downregulated (Figure 5A,B). The majority of the proteins dysregulated were specific to each treatment, whereas only 24 proteins were downregulated in both YEV and OEV treatments (Figure 5C). This observation was further confirmed by the limited clustering of the principal components between YEV and OEV treatment (Figure 5D). Heatmap analysis summarises the top 25 differentially expressed proteins (DEPs) that may potentially contribute to the phenotypic changes observed upon each treatment compared to the control (Figure 5E). The analysis also highlighted that IL‐1β‐induced activation of miR‐29b‐3p, mir‐29, CST5, NUPR1, EPAS1 and BNIP3L upstream regulators and IL‐1β‐induced inhibition of EGFR, MYC, TGF‐β, HIF1A, TEAD4 and SOX2 upstream regulators had been significantly reversed by the YEV treatment on chondrocytes (Figure 5E,F). IPA comparison analysis has identified canonical pathways and upstream regulators significantly dysregulated upon the YEV and OEV treatment. Pathway analysis indicated that YEV treatment had reversed the adverse effects of IL‐1β on chondrocytes by upregulating wound healing signalling, CLEAR signalling, and autophagy pathways and upregulated or reversed the in response to on IL‐1β induced chondrocytes. On the other hand, YEV treatment has decreased IL‐1β mediated effect on oxidative phosphorylation, necroptosis signalling, Protein Kinase A signalling, PI3K/AKT signalling and ERK/MAPK signalling (Figure 5G).

### 3.6 YEV Treatment Promotes Cartilage Regeneration in the DMM Mice Model

**FIGURE 6:**
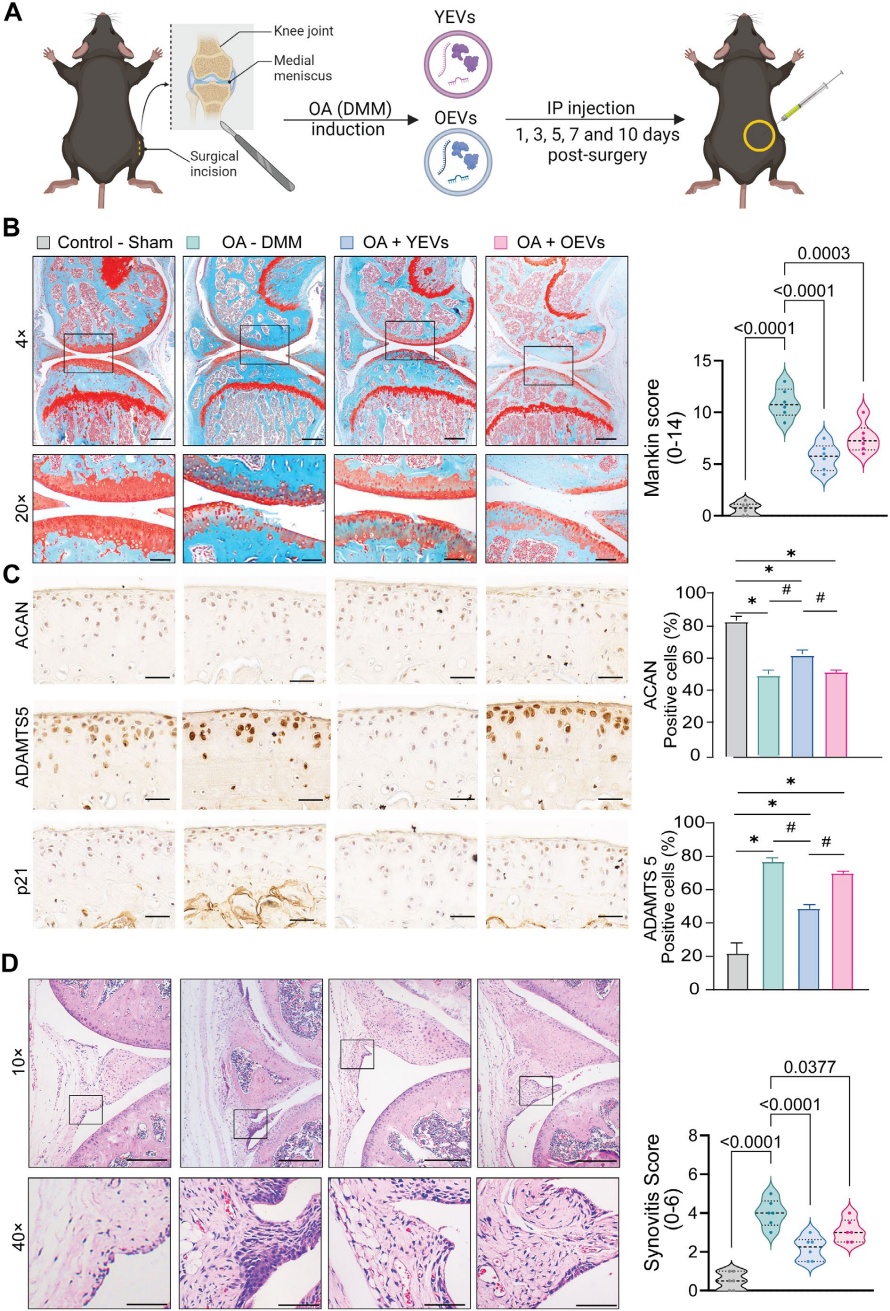
YEV alleviates the OA progression in the DMM mouse model. (A) Schematic depicting the experimental procedure for in vivo experiment. (B) Safranin O/fast green staining of knee joints of mice that underwent destabilisation of the medial meniscus (DMM) surgery or sham, with YEV and OEV treatment group. Scale bar = 100 μm. Mankin score for knee joints from all four groups. (C) ACAN, ADAMTS5 and p21 immunostaining of representative knee joint from control and DMM mice with/without EVs. Scale bar = 50 μm. ACAN and ADAMTS5 immunostaining quantitative analysis of articular cartilage. (D) Representative of H&E staining of synovial tissue from all groups and quantitative analysis of synovium score. Data represented as mean ± SD (n = 6); *compared to control, *p* ≤ 0.05, #compared to DMM, *p* ≤ 0.05.

To evaluate the therapeutic efficacy of YEVs and OEVs in OA, mice with DMM surgery‐induced post‐traumatic OA received intra‐peritoneal injections of EVs on days 1, 3, 5 and 10 following DMM surgery, as illustrated in Figure 6A. After 8 weeks, DMM surgery led to cartilage damage as determined by Safranin O/fast green staining and modified Mankin Score (6.2) in mice compared to the sham group (Figure 6B). Intra‐peritoneal injection of YEVs into DMM knees, starting immediately after the surgery, significantly improved the morphological and structural features of articular cartilage compared to OEVs. To further examine the effect of OEVs and YEVs on cartilage matrix synthesis and degradation, we performed immunostaining for aggrecan (ACAN) and A Disintegrin and Metalloproteinase with Thrombospondin Motifs 5 (ADAMTS5) and senescence marker p21. DMM surgery downregulated the expression of ACAN in articular cartilage; however, there is a significant increase in the levels of ACAN in DMM mice treated with YEV compared to the DMM group (Figure 6C). DMM mice treated with OEV could not significantly increase the expression of ACAN when compared to the DMM group. The expression of degradative marker ADAMTS5 and senescence marker p21 were strongly increased after DMM surgery. The YEV treatment significantly decreased the expression of ADAMTS5, p21 when compared to DMM and DMM mice treated with the OEV group. To investigate the effect of YEV and OEV treatment further, we performed H&E staining of the knee joints to study synovial inflammation. DMM mice showed increased inflammatory cell infiltration in synovium compared to the sham group, but OEV and YEV treatment significantly attenuated the synovitis scores (Figure 6D).

Taken together, these findings indicate that YEV treatment in the DMM model of OA effectively prevents the erosion of cartilage after DMM surgery of the OEVs.

## 4 Discussion

Ageing significantly impacts disease susceptibility and progression. The ageing process often involves the gradual loss of tissue homeostasis and regenerative capacity. This contributes to the development of degenerative diseases, including OA [34]. Moreover, the ageing process is associated with a decline in the regenerative capacity of MSCs, which play a pivotal role in maintaining joint homeostasis. As individuals age, there is a reduction in the number and functional efficiency of MSCs, impacting their ability to participate in tissue repair processes. This reduction in MSC functionality may compromise the intrinsic repair mechanisms of joint tissues, thereby contributing to the onset and progression of OA [33, 34].

The accumulation of senescent cells plays a significant role in the development and progression of age‐associated disorders like OA. The pronounced presence of senescent chondrocytes in aged human OA cartilage implies a crucial pathological mechanism in OA progression [35]. The senescence process is tightly regulated by the activation of specific tumour suppressor pathways, namely the p53/p21^WAF1/CIP1^ and p16^INK4A^/pRB pathways, which induce cell cycle arrest, thereby halting cellular proliferation [36]. Chondrocytes that express p16 demonstrate terminal differentiation into a hypertrophic phenotype and regulate the expression of matrix remodelling metalloproteinases MMP1 and MMP13 [36]. Concurrently, elevated p21 levels can lead to aberrant differentiation, limiting cellular self‐renewal capabilities, inducing apoptosis, and triggering DNA damage responses [37]. Previous research has shown a correlation between the severity of OA and the elevated levels of the senescence markers p16INK4a, p21 and p53 in addition to the DNA damage marker γH2AX [38]. Our study aligns with these observations, revealing that aged mouse cartilage not only exhibits a reduction in proteoglycan content but also an overexpression of senescence markers p16 and p21. Various studies have demonstrated that the senescent chondrocytes reduced the ability of cartilage to regenerate by impeding proliferation, promoting senescence and suppressing chondrogenic differentiation of BMSCs [39]. Moreover, we confirmed that BMSCs harvested from aged mice display a pronounced senescent phenotype, as evidenced by increased SA‐β‐gal staining, upregulation of senescence‐associated genes, reduced proliferation rates and a decline in cellular energy metabolism compared to BMSCs from younger mice. These findings underscore the association between senescence markers and OA severity, highlighting their impact on the disruption of cartilage homeostasis.

The influence of age‐associated changes in BMSCs on the composition and therapeutic potential of EV remains to be fully elucidated. It is important to explore whether EVs from young BMSCs demonstrate enhanced efficacy in promoting cartilage repair compared to their older counterparts. Given this background, we have evaluated the therapeutic effects of EVs derived from young and old BMSCs on OA in this study. Our findings revealed that EVs isolated from young and old BMSCs exhibited similar size and marker expression characteristics. Notably, EVs from young BMSCs demonstrated superior capabilities in enhancing cartilage repair and reducing senescence markers both in vitro and in vivo, suggesting that the cargo within EVs plays a critical role in cartilage repair mechanisms [40]. This finding underlines the necessity for additional research to decipher the precise action mechanisms of EVs in OA chondrocytes. Previous studies have already laid some groundwork in this domain. For instance, BMSC‐EVs isolated from young mice were found effective in extending the lifespan and mitigating senescence markers in Ercc1^−/Δ^ progeroid mice [21]. Similarly, EVs sourced from young adipose‐derived MSCs significantly enhanced ageing‐related parameters and reduced markers of oxidative stress, inflammation and senescence in muscle and kidney tissues. Additionally, these EVs reshaped the metabolome to resemble more with the young mouse than naturally ageing in a mouse model [41]. Furthermore, BMSC‐EVs have been reported to be effective in numerous preclinical OA models [42, 43, 44]. He and colleagues illustrated that EVs from young BMSCs efficiently facilitated cartilage repair, stimulated extracellular matrix synthesis and alleviated knee pain in OA rats, which was achieved through the upregulation of the COL2A1 protein and the downregulation of the MMP13 protein within the cartilage [43].

In the present study, we found that EVs from young BMSCs were particularly effective in decreasing senescence marker gene expression in IL‐1β‐induced chondrocytes and enhancing cellular energy metabolism, in contrast to EVs from older BMSCs. Notably, stimulated YEVs exhibited an enhanced capacity to protect against cellular senescence induction, as evidenced by lower expression of SASP markers, including p16, p21, IL6 and CCL2. The SASP is recognised as a key driver of prolonged cellular senescence, which significantly contributes to the pathogenesis of OA [45]. Inhibiting chondrocyte senescence is, therefore, a promising strategy to mitigate OA progression. It is well documented that the OA progression is marked by chondrocyte apoptosis, inflammation and matrix disintegration that is predominantly induced by an imbalance between catabolic and anabolic metabolisms [46]. Production of essential ECM components is crucial for proper cartilage regeneration in OA [47]. Our findings indicate that the YEVs have a pronounced effect in countering IL‐1β‐induced proteoglycan reduction in bovine cartilage explants, in contrast to EVs from older BMSCs. Moreover, in line with the induced expression of anabolic genes, YEVs have downregulated genes involved in hypertrophic differentiation and degradation, whereas OEV treatment showed insufficient chondroprotective effects on IL‐1β induced chondrocytes. Further in vivo study indicated that injection of YEVs alleviated catabolic changes by inhibiting the expression of ADAMTS5, p21, thus enhancing the chondrogenesis of cartilage, and consequently ameliorated cartilage degradation in a mouse DMM‐induced OA model, but OEVs had an inferior therapeutic effect. This finding provides evidence that the age of the parental BMSCs and potentially the composition of their EV cargo, such as microRNAs, may influence the therapeutic efficacy of EVs in OA [48]. MicroRNAs are likely one of many functional molecules which change with age [49]. Additional investigation is warranted to explore the disparities in cargo between YEVs and OEVs, the mechanisms governing their release from parental cells, and their subsequent impact on recipient cells.

To further elucidate mechanisms behind the advanced chondroprotective effects of YEVs on OA, we employed proteomic analyses to identify potential targets of YEVs and OEVs. Chondrocyte death in OA is primarily driven by defective autophagy, mitochondrial dysfunction, increased oxidative stress, and impaired lysosomal function in aged and OA‐affected cartilage correlates with elevated chondrocyte apoptosis [50, 51, 52, 53]. Promoting autophagy in chondrocytes by modulating intracellular metabolic processes has been shown in previous studies to potentially slow down the progression of OA, thereby maintaining chondrocyte viability and delaying cell senescence [25, 54, 55]. Our study demonstrated that YEVs dramatically activated the coordinated lysosomal expression and regulation (CLEAR) signalling pathway in IL‐1β treated chondrocytes, which promotes the expression of genes encoding lysosomal and autophagy‐related proteins. These findings support the notion that promoting autophagy in chondrocytes through the modulation of intracellular metabolic pathways can slow OA progression, thereby preserving chondrocyte viability and postponing senescence [56]. The proteomics analysis also showed the activation of miR‐29b‐3p, mir‐29, CST5, NUPR1, EPAS1 and BNIP3L upstream regulators after IL‐1β induction. These regulators play significant roles in the pathophysiology of OA by influencing ECM homeostasis, cartilage degradation, inflammatory responses and chondrocyte survival [57, 58, 59]. Dysregulation of these miRNAs and proteins can exacerbate the progression of OA. However, the YEV treatment reversed the IL‐1β‐induced inhibition of EGFR, MYC, TGF‐β, HIF1A, TEAD4 and SOX2 upstream regulators [60]. These regulators play important roles in various aspects, such as EGFR in cell proliferation and survival; TGF‐β signalling is critical for cartilage homeostasis [61]; in the hypoxic environment of OA cartilage, HIF1A plays a dual role in maintaining chondrocyte survival and function [62], TEAD4 in cell proliferation and differentiation, SOX2 is crucial for maintaining chondroprogenitor cell function which aligns with our results indicating YEVs, significantly reducing cartilage degradation, inflammation and senescence and also improved chondroprotection. These findings underscore the complexity of OA pathophysiology and the critical roles of various signalling pathways and transcription factors.

Our study provides insights into potential therapeutic targets by elucidating the impacts of EGFR, MYC, TGF‐β, HIF1A, TEAD4 and SOX2. Another major pathway identified in this study was the PTEN/P13K/AKT pathway. The PTEN‐modulated PI3K/Akt signalling pathway has been extensively studied because of its crucial involvement in physiological processes and disease, including OA [63, 64]. While previous studies in the PI3K/AKT pathway in OA has been mixed, suggesting pro‐catabolic and autophagy‐inhibiting effects in chondrocytes, our findings align with studies like those by Xie et al. which provide genetic evidence supporting the chondroprotective effects of PTEN‐mediated Akt inhibition [63]. This process appears crucial for maintaining cartilage homeostasis and mitigating OA progression by preventing oxidative stress‐induced cellular senescence. Our results further underscore the efficacy of YEVs in modulating these pathways, acting as dual‐function anti‐senescence agents that downregulate pro‐senescent PI3K/AKT and upregulate anti‐senescent PTEN pathways. These findings align with the current trends in regenerative therapeutics for OA which aims to alleviate symptoms and reverse degenerative processes [4, 65]. For example, senolytics are a novel class of anti‐senescence drugs that have shown promising results in animal models through indiscriminately targeting and eliminating senescent cell via apoptosis [63, 66]. Although senolytics like the p53/MDM2 interaction inhibitor UBX0101 have shown potential in reducing oxidative stress in aged murine models, the diminished regenerative capacity in older animals compared to younger ones highlights the complexity of senescence as a therapeutic target [67]. The dual nature of senescent cells which can also support tumour resistance and tissue repair, suggests that a more nuanced approach like that provided by YEVs might be beneficial [63, 68]. Furthermore, the use of platelet‐rich plasma (PRP) has become increasingly popular in the fields of anti‐aging and tissue regeneration, especially in OA [69, 70, 71]. PRP treatments have demonstrated the potential to rejuvenate cellular functions such as proliferation and differentiation, particularly in senescent cells from aged environments. This has been widely studied in both preclinical and clinical arena for OA treatment [69, 70, 71, 72, 73, 74]. However, the effectiveness of PRP is debated, attributed to the lack of standardized dosing and variations in formulations, particularly regarding leukocyte concentration which might affect therapeutic outcomes [75, 76]. Similarly, the manufacturing and regulatory pathways for EVs are less established compared to more traditional therapies, presenting challenges in standardization and quality control which limit the clinical translatability of EV therapeutics and their ability to meet future patient demands [77, 78]. These findings emphasize the importance of the senescence state of parental cells and the composition of their EV cargo in determining therapeutic efficacy. Targeting cellular senescence and metabolic pathways may therefore be a promising strategy to halt OA progression. To fully leverage the regenerative potential of YEVs and develop innovative treatments for preserving joint health, further research is crucial. Furthermore, more pre‐clinical studies are needed to identify the ideal large‐scale production of YEVs and therapeutic windows to intervene before clinical practice outpaces sound scientific data.

## 5 Conclusion

In conclusion, this study underscores the significance of donor age in the therapeutic application of BMSC‐derived EVs for OA management. Findings reveal that EVs from younger donors not only exhibit superior chondroprotective abilities and mitigate cellular senescence more effectively in vitro but also demonstrate enhanced efficacy in vivo by decelerating OA progression and alleviating synovitis in a mouse model. These outcomes suggest that integrating donor age into the selection criteria for EV‐based therapies could lead to more personalised and effective interventions for OA patients. This age‐specific approach could potentially revolutionise current treatment paradigms and provide a pathway for targeted therapies that harness the regenerative properties of young donor‐derived EVs to combat the debilitating effects of OA.

## Author Contributions

**Shital Wakale:** conceptualisation, data curation, methodology, writing – original draft. **Yang Chen:** conceptualisation, data curation, methodology, writing – original draft. **Antonia Rujia Sun:** data curation, formal analysis, methodology, writing – original draft and editing, visualisation. **Chamikara Liyanage:** formal analysis, methodology. **Jennifer Gunter:** formal analysis, methodology. **Jyotsna Batra:** project administration, resources. **Ross Crawford:** supervision, funding acquisition. **Hongxun Sang:** supervision, resources. **Indira Prasadam:** conceptualisation, supervision, funding acquisition, writing – review and editing.

## Conflicts of Interest

The authors declare no conflicts of interest.

## Supporting information


**Figure S1:** Characterisation of young and old bone marrow mesenchymal stem cells (BMSCs). (A) BMSC shows spindle‐like morphology. Scale bar 100 μm. Scale bar 100 μm. Osteogenic, adipogenic and chondrogenic differentiation of young and old BMSCs were assessed on 14 days by alizarin red, oil red and toluidine blue respectively. Scale bar 100 μm. Data represented as mean ± SD, *p ≤ 0.05; ns, not significant (*p* > 0.05).
**Figure S2:** Effect of young and old EV treatment on cell proliferation (A) MTT assay was used to determine the proliferation rate of chondrocytes after the treatment with young and old BMSC‐derived EVs for 24 h. Chondrocytes cultured in complete were used as controls. ***p < 0.0001, ****p < 0.0001 compared to control, determined by *t*‐test (*n* = 3). Data represented as mean ± SD.

**Table S1.** Target gene primer sequences for PCR.

## Data Availability

All data supporting the findings of this study are available within the paper and its Supporting Information. Primer sequences are provided in Table S1.
