# Supplementary material for: Comparative Analysis of the Therapeutic Potential of Extracellular Vesicles Secreted by Aged and Young Bone Marrow‐Derived Mesenchymal Stem Cells in Osteoarthritis Pathogenesis
Source: Cell Prolif. 2024 Dec 20;58(4):e13776. doi: 10.1111/cpr.13776 (PMC11969246; doi:10.1111/cpr.13776)
Supplement: Supplementary file 1 — Figure S1: Characterisation of young and old bone marrow mesenchymal stem cells (BMSCs). (A) BMSC shows spindle‐like morphology. Scale bar 100 μm. Scale bar 100 μm. Osteogenic, adipogenic and chondrogenic differentiation of young and old BMSCs were assessed on 14 days by alizarin red, oil red and toluidine blue respectively. Scale bar 100 μm. Data represented as mean ± SD, *p ≤ 0.05; ns, not significant (p > 0.05). Figure S2: Effect of young and old EV treatment on cell proliferation (A) MTT assay was used to determine the proliferation rate of chondrocytes after the treatment with young and old BMSC‐derived EVs for 24 h. Chondrocytes cultured in complete were used as controls. ***p < 0.0001, ****p < 0.0001 compared to control, determined by t‐test (n = 3). Data represented as mean ± SD. [file CPR-58-e13776-s002.pdf]

Supplemental Figures

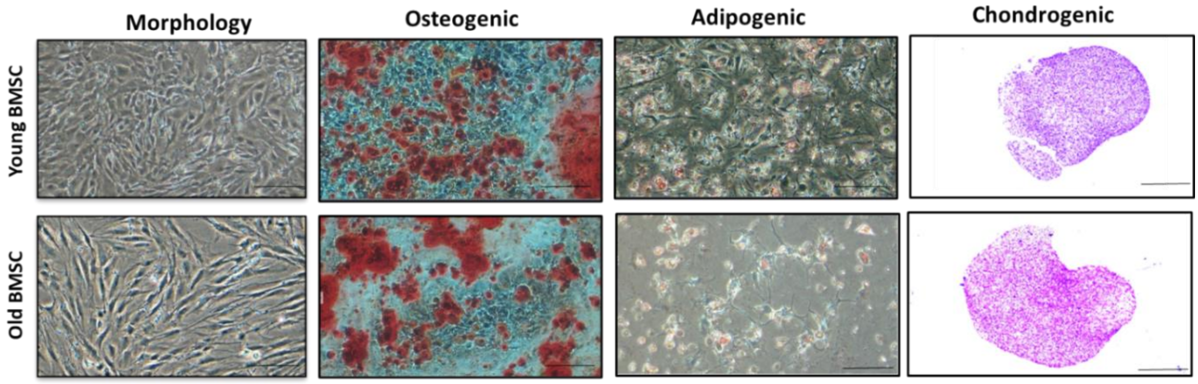

Supplemental figure S1.
Figure 1. Characterisation of young and old bone marrow mesenchymal stem cells (BMSCs). (A) BMSC shows spindle-like morphology. Scale bar 100 µm. Scale bar 100 µm. Osteogenic, adipogenic and chondrogenic differentiation of young and old BMSCs were assessed on 14 days by alizarin red, oil red and toluidine blue respectively. Scale bar 100 µm.

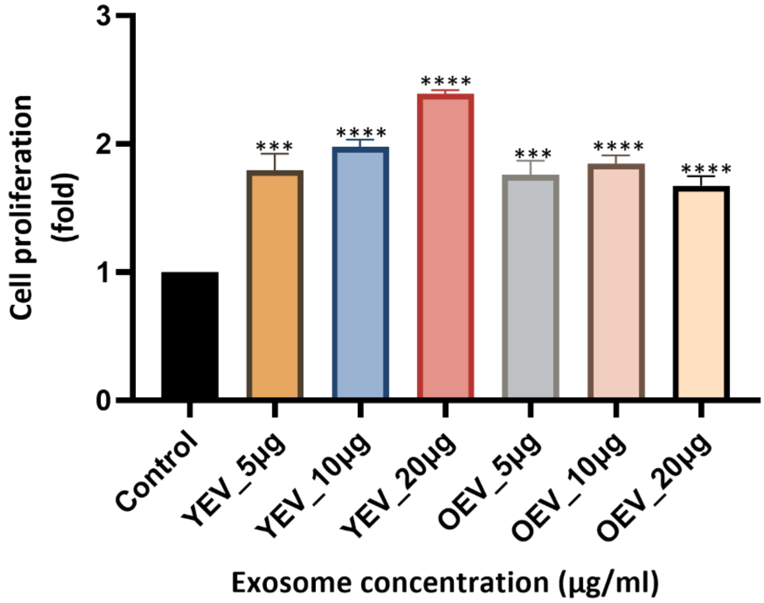

Supplemental figure S2.

Figure 2. Effect of young and old EV treatment on cell proliferation (A) MTT assay was used to determine the proliferation rate of chondrocytes after the treatment with young and old BMSC-derived EVs for 24 hours. Chondrocytes cultured in complete were used as controls. *** $p<0.0001$ **** $p<0.0001$ compared to control, determined by t-test (n=3).
