# Supplementary material for: Comparative Analysis of the Therapeutic Potential of Extracellular Vesicles Secreted by Aged and Young Bone Marrow‐Derived Mesenchymal Stem Cells in Osteoarthritis Pathogenesis
Source: Cell Prolif. 2024 Dec 20;58(4):e13776. doi: 10.1111/cpr.13776 (PMC11969246; doi:10.1111/cpr.13776)
Supplement: Supplementary file 2 — Table S1. Target gene primer sequences for PCR. [file CPR-58-e13776-s001.pdf]

Supplemental Table S1. Target gene primer sequences for PCR

Target Gene	Forward Primer Sequence	Reverse Primer Sequence
Mouse Primers		
CCL2	GCATCCACGTGTTGGCTCA	CTCCAGCCTACTCATTGGGATCA
CXCL2	CCTGGTTCAGAAAATCATCCA	CTTCCGTTGAGGGACAGC
GAPDH	AAGGTCATCCCAGAGCTGAA	CTGCTTCACCACCTTCTTGA
IL6	TGAGAAAAGAGTTGTGCAATGG	GGTACTCCAGAAGACCAGAGG
LMNB1	GGGAAGTTTATTCGCTTGAAGA	ATCTCCCAGCCTCCCATT
P16	AATCTCCGCGAGGAAAGC	GTCTGCAGCGGACTCCAT
P21	TTGCCAGCAGAATAAAAGGTG	TTTGCTCCTGTGCGGAAC
PAI1	ACGCCTGGTGCTGGTGAATGC	ACGGTGCTGCCATCAGACTTGTG
TIMP1	CACACCAGAGCAGATACCATGA	GGGGAACCCATGAATTTAGCC
TNF-α	ATGAGAAGTTCCCAAATGGC	CTCCACTTGGTGGTTTGCTA
Human Primers		
ACAN	GTGCCTATCAGGACAAGGTCT	GATGCCTTTCACCACGACTTC
ADAMTS4	TATCCTCAGGGTCCGTTCCT	TCTGGAGACTCCGTTCCATCT
ADAMTS5	GAACATCGACCAACTCTACTCCG	CAATGCCCACCGAACCATCT
CCL2	TCGCCTCCAGCATGAAAGTC	GGTGACTGGGGCATTGATTG
COL2A1	TGGACGATCAGGCGAAACC	GCTGCGGATGCTCTCAATCT
COMP	CAAGGCCAACAAGCAGGTTT	GTTGATGCACACGGAGTTGG
GAPDH	AAGGTCATCCCAGAGCTGAA	CTGCTTCACCACCTTCTTGA
IL6	TCTCCACAAGCGCCTTCG	CTCAGGGCTGAGATGCCG
Ki67	GTTCTTCCCTGGCACTGTTGAC	CAGACCCATTTACTTGTGTTGGA
LMNB	GAGAGCAACATGATGCCCAAGTG	GTTCTTCCCTGGCACTGTTGAC
p16	GGGTTTTCGTGGTTCACATCC	CTAGACGCTGGCTCCTCAGTA
p21	TGTCCGTCAGAACCCATGC	AAAGTCGAAGTTCCATCGCTC
PCNA	GCGTGAACCTCACCAGTATGT	TCTTCCCCTTAGTTAATGAT
PRG	CAGAGGTCTCTACTCCAACTACC	AGTCATTTCAGGTTTAGTCGCTG
SOX5	CAGCCAGAGTTAGCACAATAGG	CTGTTGTTCCCGTCGGAGTT
SOX6	TACCTCTACCTCACCACATAAGC	ACATCGGCAAGACTCCCTTTG
Trp53	CCTCAGCATCTTATCCGAGTGG	TGGATGGTGGTACAGTCAGAGC
